# In vitro and in vivo studies on exogenous polyamines and α-difluoromethylornithine to enhance bone formation and suppress osteoclast differentiation

**DOI:** 10.1007/s00726-024-03403-8

**Published:** 2024-06-27

**Authors:** Chien-Ching Lee, Chia-Chun Chuang, Chung-Hwan Chen, Yuan-Pin Huang, Chiao-Yi Chang, Pei-Yi Tung, Mon-Juan Lee

**Affiliations:** 1 Department of Anesthesia, An Nan Hospital, China Medical University, Tainan, 70965 Taiwan; 2 Department of Medical Science Industries, Chang Jung Christian University, No.1, Changda Rd., Gueiren District, Tainan, 711301 Taiwan; 3 Department of Orthopedics, Kaohsiung Municipal Ta-Tung Hospital, Kaohsiung, 80145 Taiwan; 4 Department of Orthopedics, Kaohsiung Medical University Hospital, Kaohsiung Medical University, Kaohsiung, 80756 Taiwan; 5 Orthopaedic Research Center, Kaohsiung Medical University, Kaohsiung, 80756 Taiwan; 6 Department of Orthopedics, College of Medicine, Kaohsiung Medical University, Kaohsiung, 80756 Taiwan; 7 Regenerative Medicine and Cell Therapy Research Center, Kaohsiung Medical University, Kaohsiung, 80756 Taiwan; 8 Department of Cosmetics and Fashion Styling, Cheng Shiu University, Kaohsiung, 83347 Taiwan; 9 Department of Bioscience Technology, Chang Jung Christian University, Tainan, 711301 Taiwan

**Keywords:** Polyamines, α-difluoromethylornithine (DFMO), Osteogenic differentiation, Osteoclastogenic differentiation, Ovariectomized rat, Osteoporosis

## Abstract

Exogenous polyamines, including putrescine (PUT), spermidine (SPD), and spermine (SPM), and the irreversible inhibitor of the rate-limiting enzyme ornithine decarboxylase (ODC) of polyamine biosynthesis, α-difluoromethylornithine (DFMO), are implicated as stimulants for bone formation. We demonstrate in this study the osteogenic potential of exogenous polyamines and DFMO in human osteoblasts (hOBs), murine monocyte cell line RAW 264.7, and an ovariectomized rat model. The effect of polyamines and DFMO on hOBs and RAW 264.7 cells was studied by analyzing gene expression, alkaline phosphatase (ALP) activity, tartrate-resistant acid phosphatase (TRAP) activity, and matrix mineralization. Ovariectomized rats were treated with polyamines and DFMO and analyzed by micro computed tomography (micro CT). The mRNA level of the early onset genes of osteogenic differentiation, Runt-related transcription factor 2 (Runx2) and ALP, was significantly elevated in hOBs under osteogenic conditions, while both ALP activity and matrix mineralization were enhanced by exogenous polyamines and DFMO. Under osteoclastogenic conditions, the gene expression of both receptor activator of nuclear factor-κB (RANK) and nuclear factor of activated T-cells, cytoplasmic 1 (NFATc1) was reduced, and TRAP activity was suppressed by exogenous polyamines and DFMO in RAW 264.7 cells. In an osteoporotic animal model of ovariectomized rats, SPM and DFMO were found to improve bone volume in rat femurs, while trabecular thickness was increased in all treatment groups. Results from this study provide in vitro and in vivo evidence indicating that polyamines and DFMO act as stimulants for bone formation, and their osteogenic effect may be associated with the suppression of osteoclastogenesis.

## Introduction

Osteoporosis is a silent disease of gradual bone loss that occurs in post-menopausal women and the elderly and is often overlooked and undertreated (Bouvard et al. 2021). Patients are not aware of the progress of the disease until osteoporotic fracture occurs. Currently, no treatment can completely reverse osteoporosis, and only a limited selection of medication is available (Kim et al. 2021). Since the approval of teriparatide by the U.S. Food and Drug Administration (FDA) in 2002, no other bone-stimulating drug was launched until the discovery of romosozumab, the anti-sclerostin antibody (Cosman et al. 2016). Alternatives to teriparatide and romosozumab are drugs that passively inhibit bone loss without inducing bone formation. As the elderly population increases worldwide, we are fighting a losing battle against osteoporosis.

Polyamines are the organic polycations that prevail in all organisms, with putrescine (PUT), spermidine (SPD), and spermine (SPM) being the most common polyamines in mammalian cells (Sagar et al. 2021). Polyamines exhibit antioxidative and anion- and cation-binding properties and are involved in numerous cell growth and differentiation processes (LØVaas 1996; Sagar et al. 2021). Described as mysterious modulators of cellular functions (Igarashi and Kashiwagi 2000), polyamines are implicated in reversing B cell senescence (Zhang et al. 2019), protecting against diet-induced obesity (Choksomngam et al. 2021), and regulation of bone homeostasis (Zhang et al. 2023). In musculoskeletal tissues, polyamines are associated with the growth and development of bone and cartilage (Matsui-Yuasa et al. 1985; Vittur et al. 1986). Animal studies performed with ovariectomized mice as the osteoporotic model indicate that spermidine (SPD) and spermine (SPM), two of the common mammalian polyamines, prevented bone loss by disrupting osteoclast activities (Yamamoto et al. 2012). SPM was also found to promote osteogenic differentiation in goat adipose tissue-derived mesenchymal stem cells (ADSCs) (Tjabringa et al. 2006, 2008). Patients with Snyder-Robinson Syndrome (SRS), an X-linked intellectual disability disorder caused by a mutation in spermine synthase, suffer from osteoporosis in which osteoblasts and osteoclasts are severely depleted (Albert et al. 2015). Recently, modulation of gut microbiota was reported to regulate bone remodeling, and treatment with probiotics has become a new approach to osteoporosis therapy (Lyu et al. 2023). Warmth and warm microbiota transplantation enhances bacterial polyamine biosynthesis, resulting in higher total polyamine levels and increased bone strength in mice (Chevalier et al. 2020).

It was previously demonstrated that exogenous PUT, SPD, and SPM, as well as α-difluoromethylornithine (DFMO), the irreversible inhibitor of the rate-limiting ornithine decarboxylase (ODC) in the polyamine biosynthetic pathway, reciprocally regulate osteogenic and adipogenic gene expression as well as terminal differentiation in human bone marrow-derived mesenchymal stem cells (hBMSCs) (Lee et al. 2013; Tsai et al. 2015). Both exogenous polyamines and DFMO act through suppression of ODC, resulting in a decrease in the level of intracellular polyamines. In this study, the osteogenic activities of polyamines and DFMO were examined in human osteoblasts (hOBs), murine macrophage cell line RAW 264.7, and an ovariectomized rat model to elucidate further the correlation of the polyamine biosynthetic pathway to bone formation.

## Methods

### Cell culture

Human OBs isolated from normal healthy adult human bone were provided by Cell Applications, Inc. (San Diego, CA, USA). The cells were cultured in Osteoblast Growth Medium provided by the manufacturer or in Dulbecco’s Modified Eagle Medium (DMEM), low glucose, supplemented with 10% fetal bovine serum (Gibco, Thermo Fisher Scientific, Waltham, MA, USA), 50 U/mL penicillin, and 50 mg/mL streptomycin (Invitrogen, Thermo Fisher Scientific, Waltham, MA, USA) at 37 °C and 5% CO_2_ in a humidified incubator. Murine RAW 264.7 cell line was purchased from Bioresource Collection and Research Center, Hsinchu, Taiwan, for which high-glucose DMEM with 10% fetal bovine serum, 50 U/mL penicillin, and 50 mg/mL streptomycin was used as culture medium.

### Cell treatment

Human OBs were seeded at 10^4^ cells/well in 24-well plates for alizarin red S and alkaline phosphatase (ALP) staining, and at 5 × 10^4^ cells/well in 6-well plates for total RNA extraction. Two days after seeding, hOBs were treated for 7 or 14 days with PUT, SPD, SPM, or DFMO prepared in the osteogenic induction medium (OIM), which was DMEM supplemented with 10^− 7^ M dexamethasone, 10 mM β-glycerolphosphate, and 50 µM L-ascorbate 2-phosphate. In the presence of PUT, SPD, or SPM, the differentiation medium was supplemented with an additional 1 mM aminoguanidine to inhibit bovine serum amine oxidase. RAW 264.7 cells were seeded at 5 × 10^4^ cells/well in 24-well plates for tartrate-resistant acid phosphatase (TRAP) staining and at 2 × 10^5^ cells/well in 6-well plates for total RNA extraction. Two days after seeding, osteoclastogenic differentiation was induced by culturing cells in high-glucose DMEM containing 50 ng/ml receptor activator of nuclear factor kappa-B ligand (RANKL, Abcam Limited, Cambridge, UK) for 4 days in the presence or absence of various concentrations of exogenous polyamines or DFMO.

### Real-time polymerase chain reaction

Total RNA was extracted from hOBs or RAW 264.7 cells by the PureLink™ RNA Mini Kit (Thermo Fisher Scientific, Waltham, MA, USA), and cDNA synthesis was performed using the QuantiNova™ Reverse Transcription Kit (Qiagen, Germantown, MD, USA) based on the manufacturer’s instructions. Five ng of the resulting cDNA was mixed with gene-specific primer pairs designed by OriGene, Rockville, MD, USA (Table 1), and reagents from the QuantiFast SYBR® Green PCR Kit (Qiagen, Germantown, MD, USA). The reaction mixture was subjected to initial activation for 5 min at 95 °C, followed by 40 cycles of denaturation at 95 °C for 10 s and annealing combined with extension at 60 °C for 30 s, and ending with melting curve analysis in an Applied Biosystems 7300 Real-Time PCR System equipped with Applied Biosystems Sequence Detection Software V1.2 (Life Technologies, Carlsbad, CA, USA).

**Table 1:** Sequences of real-time PCR primer pairs used in this study

Gene	Primer	Sequence	NCBI Accession number
Runx2	Forward Reverse	CCCAGTATGAGAGTAGGTGTCC GGGTAAGACTGGTCATAGGACC	NM_004348
ALP	Forward Reverse	GCTGTAAGGACATCGCCTACCA CCTGGCTTTCTCGTCACTCTCA	NM_000478
Osteopontin	Forward Reverse	CGAGGTGATAGTGTGGTTTATGG GCACCATTCAACTCCTCGCTTTC	NM_000582
Osteocalcin	Forward Reverse	CGCTACCTGTATCAATGGCTGG CTCCTGAAAGCCGATGTGGTCA	NM_199173
RANK	Forward Reverse	GCTCAACAAGGACACAGTGTGC CGCATCGGATTTCTCTGTCCCA	NM_003839
NFATc1	Forward Reverse	CACCAAAGTCCTGGAGATCCCA TTCTTCCTCCCGATGTCCGTCT	NM_172387
TRAF6	Forward Reverse	CAATGCCAGCGTCCCTTCCAAA CCAAAGGACAGTTCTGGTCATGG	NM_145803
GAPDH	Forward Reverse	GTCTCCTCTGACTTCAACAGCG ACCACCCTGTTGCTGTAGCCAA	NM_002046

### Cell staining

Cells were fixed with 4% paraformaldehyde in PBS at room temperature for 15 min, followed by incubation with 0.2% alizarin red S in ddH_2_O (pH 6.4) or Pierce 1-Step NBT/BCIP Solution (Thermo Fisher Scientific, Waltham, MA) for 20 min at room temperature to determine the level of matrix mineralization or ALP activity, respectively. The stained cells were rinsed with ddH_2_O and air-dried at room temperature before being observed under a phase‐contrast optical microscope. To quantify cell-bound alizarin red S, the stained cells were dissolved in 10% cetylpyridinium chloride for 10 min with shaking, followed by the determination of the absorbance at 570 nm, which was interpolated on a standard curve to calculate the absolute concentration of the solubilized alizarin red S. The concentration of alizarin red S was then normalized to the total amount of DNA (µg) determined by Quant-iT™ PicoGreen® dsDNA Assay Kit (Life Technologies, Carlsbad, CA, USA). To quantify ALP activity, the stained cells were dissolved in dimethyl sulfoxide (DMSO) and centrifuged at 12,000 rpm for 10 min to remove insoluble precipitates. The absorbance of the resulting DMSO-solubilized sample was measured at 570 nm. For TRAP staining, a TRAP staining kit manufactured by Takara Bio Inc. was applied in which a staining solution containing 0.05 M sodium tartrate was used. TRAP-positive RAW 264.7 cells were counted and averaged from 5 individual fields at a 100x magnification to represent the level of osteoclastogenesis.

### Osteoporotic animal model

The animal use protocol was reviewed and approved by the Institutional Animal Care and Use Committee of Chang Jung Christian University, Tainan, Taiwan, and National Laboratory Animal Center of National Applied Research Laboratories, Taipei, Taiwan, where the animal studies were performed. Female ovariectomized Sprague Dawley (SD) rats of 12-week old were purchased from BioLASCO Taiwan Co., Ltd., Taipei, Taiwan. We treated 16-week-old ovariectomized SD rats daily with 10 mM PUT, SPD, or SPM in drinking water, or with 50 mg/day DFMO by intraperitoneal injection for 6 weeks. For the treatment groups of PUT, SPD, and SPM, the volume of drinking water was recorded every day to calculate the total amount of polyamines ingested. A total of 15 ovariectomized SD rats were used in this study, which included 2 untreated ovariectomized controls and 3 for each treatment group. The animals were subjected to micro computed tomography (micro CT, SKYSCAN 1076) scanning one week before polyamine or DFMO treatment (17 weeks old), and were scanned at 0, 2, and 6 weeks during treatment, followed by a final scan at 7 weeks after the last injection, at which the SD rats were 31 weeks old. The animals were then euthanized for histological analysis.

### Statistical analysis

Statistical significance was evaluated by Student’s *t*-test. Levels of significance were expressed as significant, *p* < 0.05, and highly significant, *p* < 0.01, respectively.

## Results and discussion

### Effect of exogenous polyamines and DFMO on osteogenic gene expression in hOBs

**Fig. 1:**
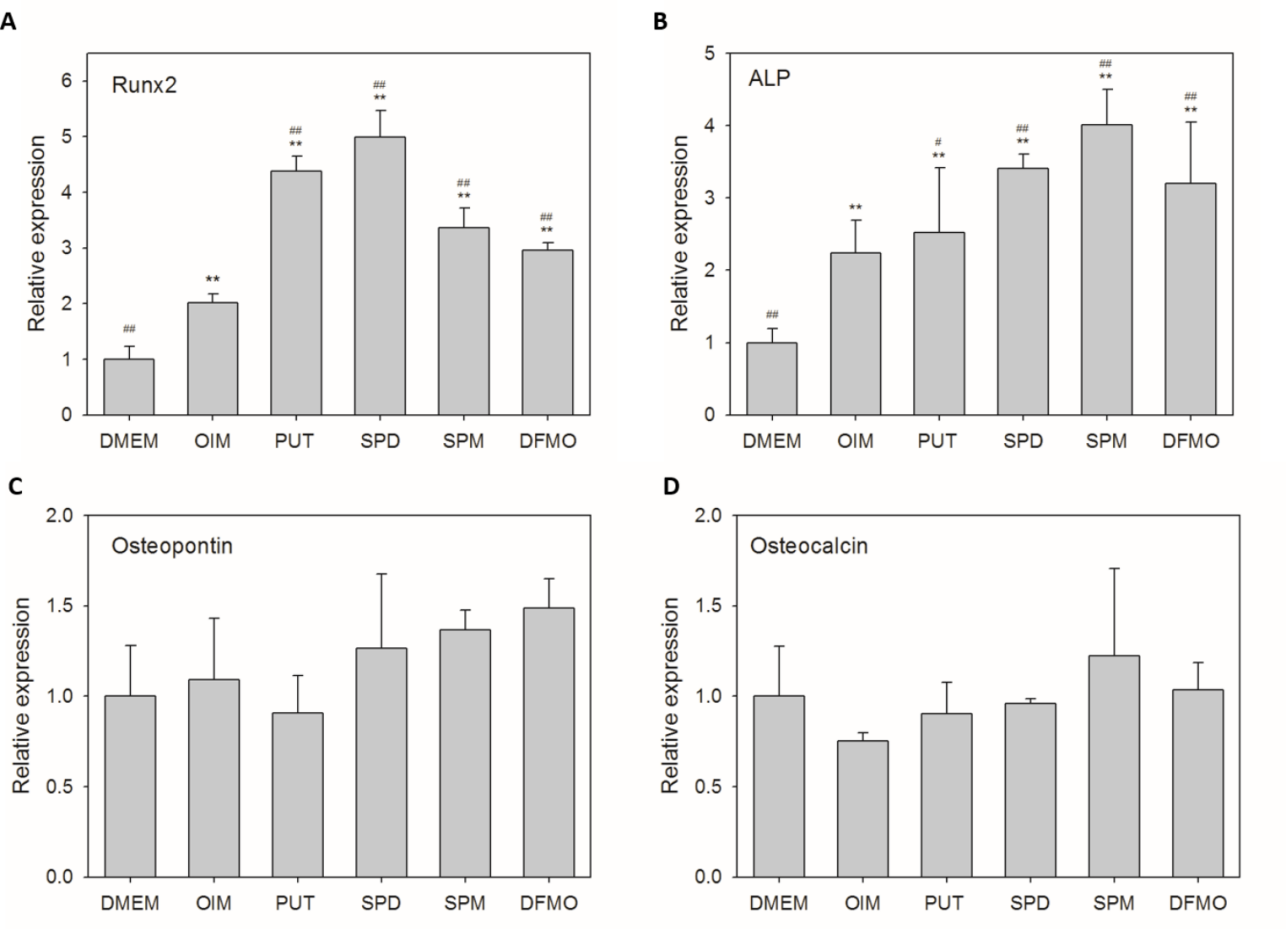
Effect of exogenous polyamines and DFMO on the osteogenic gene expression of hOBs. Human OBs were treated with 1 µM PUT, 1 µM SPD, 1 µM SPM, or 10 µM DFMO in OIM for 7 days. Relative gene expression of (**A**) Runx2, (**B**) ALP, (**C**) osteopontin, and (**D**) osteocalcin was determined by real-time PCR. Error bars represent standard deviations (*n* ≥ 3). *p < 0.05 and **p < 0.01, compared to the control (DMEM); ^#^*p* < 0.05 and ^##^*p* < 0.01, compared to OIM

We studied the effect of exogenous polyamines and DFMO on the mRNA level of representative osteogenic genes including Runx2 and ALP, the early-onset genes of osteogenic differentiation, as well as osteopontin and osteocalcin, which are late-onset genes responsible for extracellular matrix mineralization. The mRNA level of Runx2 and ALP was significantly elevated when hOBs were cultured in the presence of exogenous polyamines or DFMO for 7 days, compared to that of untreated cells and hOBs cultured in OIM alone (Fig. 1A and B). The gene expression of osteopontin and osteocalcin was unaffected by exogenous polyamines or DFMO at this stage (Fig. 1C and D), as these late-onset genes may be up-regulated after prolonged osteogenic induction.

### Effect of exogenous polyamines and DFMO on ALP activity and matrix mineralization in hOBs

**Fig. 2:**
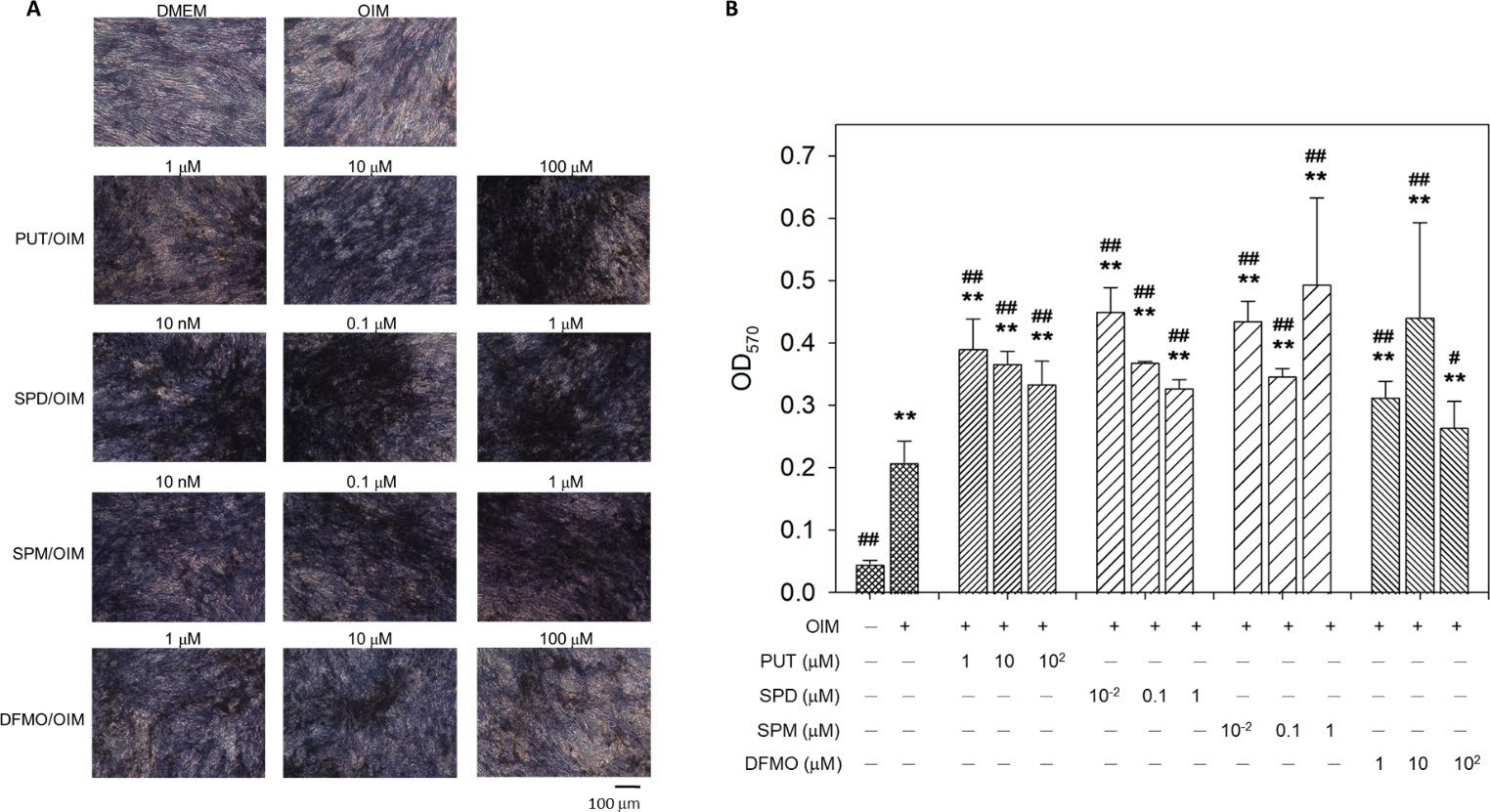
Effect of exogenous polyamines and DFMO on the ALP activity of hOBs. Human OBs were treated with various concentrations of exogenous polyamines or DFMO in OIM for 14 days, followed by reaction with Pierce 1-Step NBT/BCIP Solution (Thermo Fisher Scientific, Waltham, MA, USA) according to the manufacturer’s instructions

**Fig. 3:**
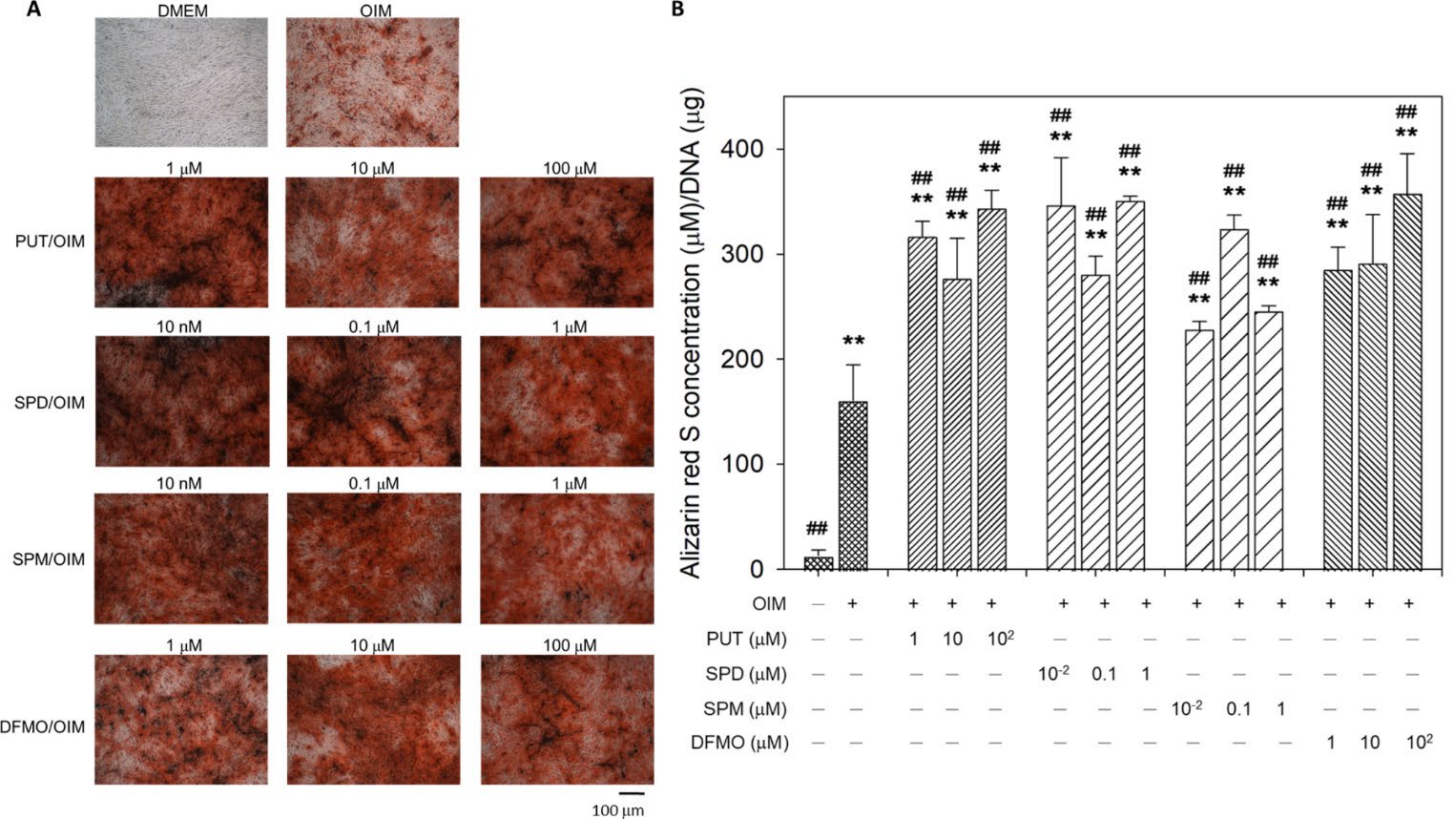
Effect of exogenous polyamines and DFMO on matrix mineralization of hOBs. Human OBs were treated with various concentrations of exogenous polyamines or DFMO in OIM for 14 days and stained with alizarin red S to determine the level of matrix mineralization

When hOBs were treated with OIM supplemented with various concentrations of polyamines or DFMO for 14 days, ALP activity was significantly enhanced compared to untreated cells and hOBs cultured in OIM alone (Fig. 2), in accordance with the gene expression profile of ALP in Fig. 1B. Furthermore, matrix mineralization was accelerated by exogenous polyamines and DFMO as well (Fig. 3). These results suggest that exogenous polyamines and DFMO may direct hOBs toward the osteoblast lineage by promoting osteogenic gene expression, ALP activity, and matrix mineralization. Nevertheless, when compared with results in the literature, it was reported that ALP activity remained unaffected in the presence of 10–1000 nM PUT, SPD, or SPM in mouse pre-osteoblastic cells MC3T3-E1 induced to differentiate osteogenically (Yamamoto et al. 2012). This discrepancy may result from the differentiation state of human osteoblasts compared to MC3T3-E1, an osteogenic induction condition in the absence of dexamethasone for MC3T3-E1, and the different time points of observation (14 d in this study vs. 21 d in the literature). On the other hand, because DFMO is the irreversible inhibitor of ODC, the enzyme for the rate-limiting step of the polyamine biosynthetic pathway, the similar effect of DFMO to exogenous polyamines on the osteogenic differentiation of hOBs suggests that both exogenous polyamines and DFMO may act on hOBs through suppression of endogenous polyamine synthesis, supporting our previous findings in hBMSCs (Lee et al. 2013; Tsai et al. 2015).

One potential connection between polyamine biosynthesis and osteoblast differentiation may be through the Sp1/Sp3 transcription factors of the specificity proteins (Sp)/Krüppel-like transcription factor family. Abnormal ossification and reduced osteocalcin gene expression were observed in Sp3-deficient mouse embryos (Göllner et al. 2001). ODC is negatively regulated by Sp3, which antagonizes activation of the ODC promoter by Sp1 and activates the promoter of liver/bone/kidney-type alkaline phosphatase (ALP) (Kumar and Butler 1997; Yusa et al. 2000). Our previous studies have shown that suppression of ODC through DFMO or the small interfering RNA against ODC resulted in increased ALP activity and up-regulation of Sp3 in hBMSCs (Tsai et al. 2015).

### Effect of exogenous polyamines and DFMO on osteoclastogenic gene expression in RAW 264.7 cells

**Fig. 4:**
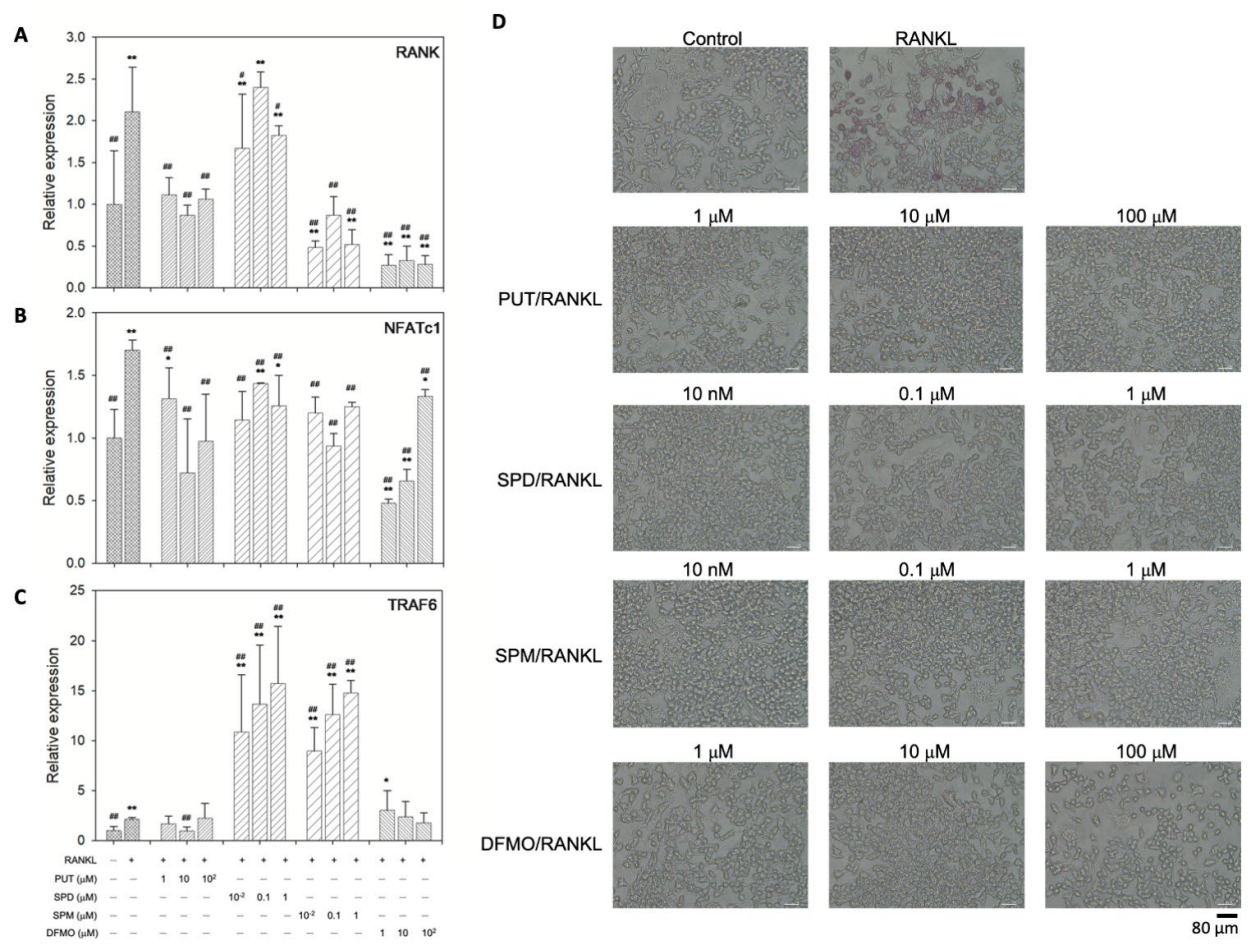
Effect of exogenous polyamines and DFMO on the osteoclastogenic gene expression of RAW 264.7 cells. RAW 264.7 cells were treated with various concentrations of exogenous polyamines or DFMO in DMEM supplemented with 50 ng/ml RANKL (RANKL medium) for 4 days. Relative gene expression of (**A**) Rank, (**B**) Nfatc1, and (**C**) Traf6 was determined by real-time PCR. (**D**) TRAP activity was determined by a TRAP staining kit as described in Materials and Methods. Error bars represent standard deviations (*n* ≥ 3). *p < 0.05 and **p < 0.01, compared to the control (DMEM); ^#^*p* < 0.05 and ^##^*p* < 0.01, compared to RANKL medium

We induced osteoclastogenic differentiation of the murine macrophage cell line RAW 264.7 with 50 ng/ml RANKL for 4 days in the presence of various concentrations of polyamines and DFMO to determine their effects on osteoclastogenic gene expression (Fig. 4A-C). In the presence of RANKL, the expression of osteoclastogenic genes, including receptor activator of nuclear factor-κB (RANK), nuclear factor of activated T-cells, cytoplasmic 1 (NFATc1), and tumor necrosis factor receptor associated factor 6 (TRAF6) increased compared to untreated controls. Under the treatment of PUT and DFMO, the expression of RANK, NFATc1, and TRAF6 decreased significantly at most concentrations tested. For SPD, however, only the expression of NFATc1 was significantly affected, and for SPM, the expression of RANK and NFATc1, but not TRAF6, was reduced significantly. The mRNA level of TRAF6 was significantly upregulated in the presence of SPD and SPM. On the other hand, we observed that the TRAP activity was suppressed in all treatment groups, indicating that osteoclastogenesis was inhibited in the presence of exogenous polyamines and DFMO (Fig. 4D). Except for cells with RANKL-only treatment, TRAP-positive mononuclear cells were barely observed in RAW 264.7 cells treated with polyamines or DFMO. The average number of TRAP-positive mononuclear cells in RAW 264.7 cells treated only with RANKL was 32 ± 6 cells per field in a representative micrograph at 100x magnification shown in Fig. 4D, while those for polyamine- or DFMO-treated cells were 0.2 to 0.8. cells per field, an average from 5 different fields. No TRAP-positive cells were seen in the control group without RANKL treatment. These results suggest that suppression of osteoclastogenic differentiation by PUT, SPM, and DFMO may be mediated by the RANK-NFATc1 signaling pathway, but the suppressive effect of SPD on osteoclast differentiation may be caused by other mediators.

Compared with results in the literature, suppressed osteoclastogenesis by PUT, SPD, and DFMO was observed in osteoclasts derived from mouse hematopoietic stem cells (Brunner et al. 2020). In the osteoclast differentiation of mouse bone marrow-derived macrophages, PUT, SPD, and SPM down-regulated the mRNA level of NFATc1 and inhibited the RANKL-induced formation of multinucleated cells and migration of preosteoclasts (Yeon et al. 2014). In addition, Yamamoto et al. reported the inhibition of RANKL-induced osteoclast differentiation for SPD and SPM but not for PUT (Yamamoto et al. 2012). This may be due to the observation in this and our previous studies that compared to SPD and SPM, a higher concentration of PUT (up to 100 µM) is required for its osteogenic effect to be seen, and cells had a higher tolerance for PUT as well (Lee et al. 2013). The mechanism behind the inhibition of RANKL-induced osteoclastogenesis by polyamines and DFMO remained elusive. It is known that RANKL induced the transient up-regulation of the cyclin-dependent kinase inhibitor p21^WAF1/CIP1^, whose promoter contains Sp1/Sp3 binding sequences (Okahashi et al. 2001; Santini et al. 2001). On the other hand, the promoter region of NFATc1 also includes Sp1/Sp3 binding sites, suggesting that cross-talk between Sp1/Sp3, p21^WAF1/CIP1^, and NFATc1 is possible (Serfling et al. 2012). Results from these studies provide implications that polyamines and DFMO may be connected with the signal transduction of osteoclast differentiation through Sp1 and Sp3 to regulate effectors such as p21 and NFATc1 downstream of RANKL/RANK signaling.

### Effect of exogenous polyamines and DFMO on osteoporotic animal model

**Fig. 5:**
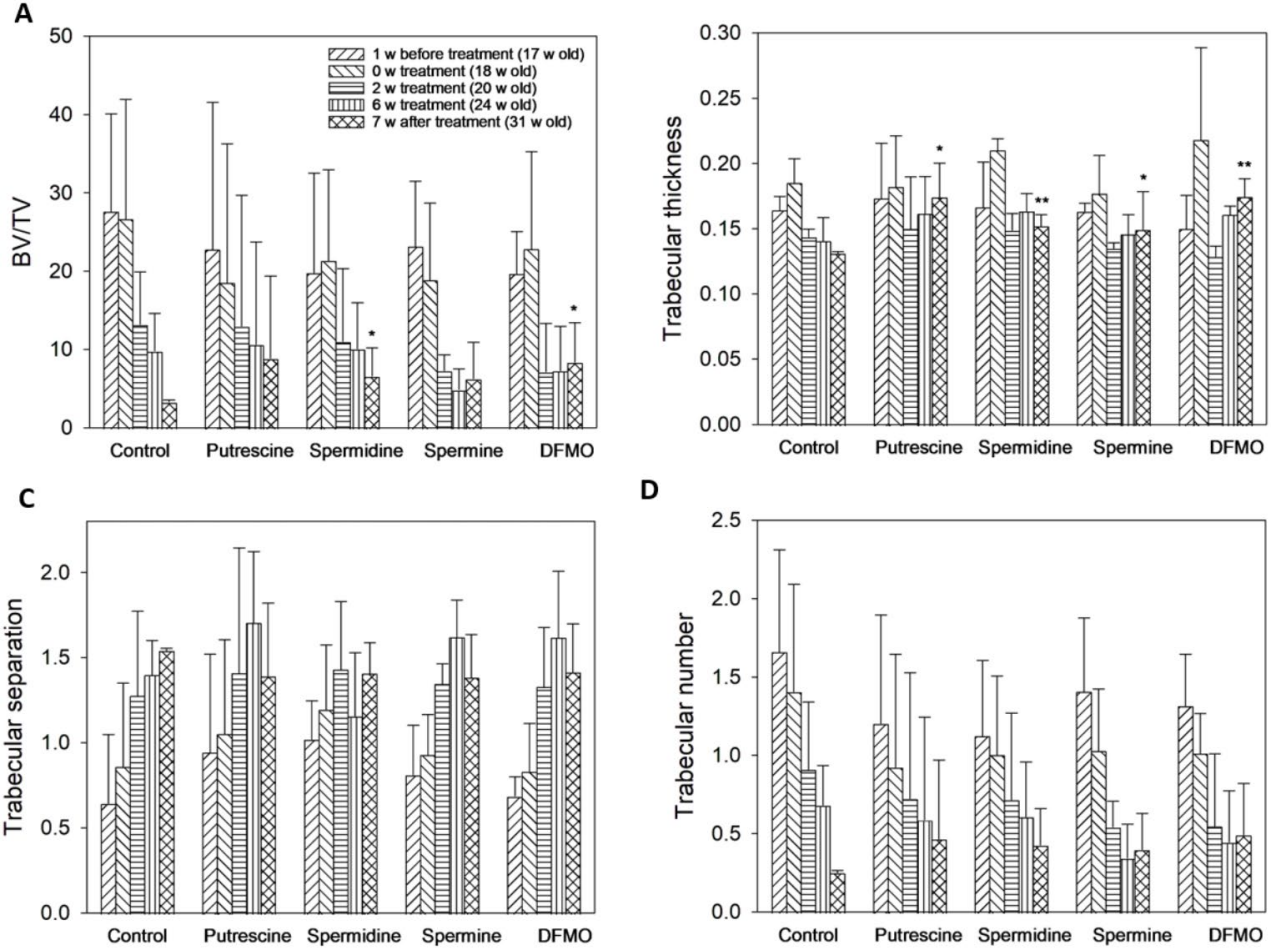
Micro CT analysis of the effect of exogenous polyamines and DFMO on ovariectomized rat model. Effect of exogenous polyamines and DFMO on (**A**) femoral bone volume, (**B**) trabecular separation, (**C**) trabecular thickness, and (**D**) trabecular number of ovariectomized SD rats were analyzed by micro CT. *p < 0.05 and **p < 0.01, compared to the ovariectomized control

**Fig. 6:**
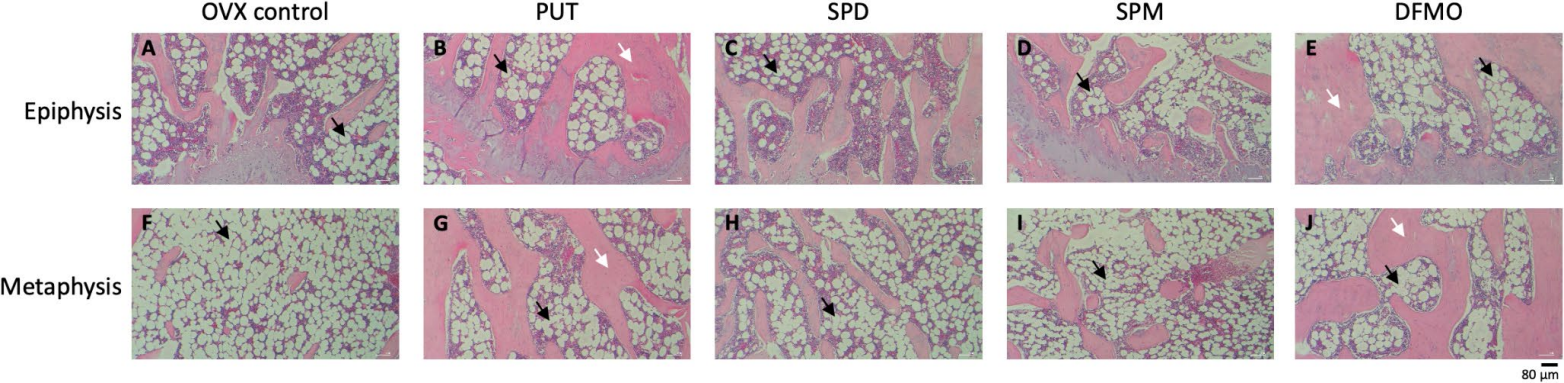
H&E staining of femur bone sections from ovariectomized rat model. Representative images of epiphysis and metaphysis were presented for the ovariectomized control and the treatment groups. Black arrow, representative area of fat cells; white arrow, representative area of trabecular tissue. Scale bar, 80 μm

The average total amount of putrescine, spermidine, and spermine ingested by each rat during 6 weeks of treatment was 1.95, 3.09, and 2.38 g, respectively, while a total of 2.10 g DFMO per rat was injected intraperitoneally. Scanning results of rat femur using micro CT indicate that 7 weeks after continuous treatment with exogenous polyamines or DFMO, bone volume was significantly elevated when ovariectomized rats were treated with spermidine or DFMO (Fig. 5A), and trabecular thickness was increased in all treatment groups (Fig. 5B). However, the difference in trabecular separation and trabecular number between treatment groups and the control remained insignificant (Fig. 5C, D). Although normal and sham control was not included in the animal study, we did find that the dosage of polyamines and DFMO applied was nontoxic to the ovariectomized SD rats. The increase in the body weight of the ovariectomized rats in both the control and treatment groups followed a similar trend (data not shown). Hematoxylin and eosin (H&E) staining results suggest that the osteoporotic animal model was established, as the trabecular bone section was populated with fat-rich tissue in untreated ovariectomized rats (Fig. 6A, F). In addition, an increase in trabecular thickness in the treatment groups can be observed in Fig. 6B-E and G-J when compared with the ovariectomized control, supporting the results from micro CT analysis (Fig. 5B). Previous studies have shown that bone volume was increased in the vertebrae of ovariectomized mice treated with 0.3 or 3 mM spermidine or spermine in drinking water for 28 consecutive days (Yamamoto et al. 2012). In ovariectomized rats, we demonstrated that not only spermidine and spermine, but also putrescine and DFMO are potential stimulants of bone formation.

## Data Availability

Data is provided within the manuscript.
